# Results of an Ultra Short Metaphyseal Filling Cementless Stem in Primary Total Hip Arthroplasty: Mean 12.1-Year Follow-up

**DOI:** 10.1007/s43465-025-01641-w

**Published:** 2025-12-05

**Authors:** Sachin R. Tapasvi, Madhav Chowdhry, Anshu Shekhar, Komal S. Tapasvi, James A. Browne, Edward J. McPherson

**Affiliations:** 1 Department of Arthroplasty and Arthroscopy, The Orthopaedic Specialty Clinic, Pune, Maharashtra India; 2 Department of Orthopaedic Surgery, Jawaharlal Nehru Medical College, Aligarh Muslim University, Aligarh, Uttar Pradesh India; 3 Department of Orthopaedic Surgery, Sancheti Institute for Orthopaedics and Rehabilitation, Pune, Maharashtra India; 4 Department of Orthopaedic Surgery, University of Virginia, Charlottesville, USA; 5 Department of Orthopaedic Surgery, David Geffen School of Medicine at UCLA, Los Angeles, USA

**Keywords:** Ultra-short stem, USS, Total hip arthroplasty, THA, Cementless, Femoral component, Proxima, Stemless, Young

## Abstract

**Introduction:**

Cementless ultra-short stems (USS), defined as implants that do not engage the diaphyseal cortex (stemless), have been designed to leave diaphyseal bone undisturbed. This study reports midterm clinical results using an USS in relatively younger patients undergoing primary total hip arthroplasty (THA).

**Methods:**

The study included 32 THA in 26 patients (8 females, 18 males) with end-stage coxarthrosis of various etiologies, with patients $\le$ 70 years of age using the USS Proxima hip stem. All cups were of cementless design with several bearing configurations and head diameters.

**Results:**

The mean age at surgery was 47.9 years (28–67 years). The mean follow-up was 12.1 years (range 10.8–13.8 years). Seven patients were classified as Dorr A femoral bone type, 18 were Dorr B, and 7 were Dorr C. Mean Harris Hip Score (HHS) improvement was 39.3 points from preoperative (*p* = 0.0001), with 10-year HHS averaging 88. There were two stem failures. One was due to a periprosthetic joint infection, which was removed at 3 years. The other stem was aseptically loose and revised at 8 years. Stem survivorship was 93.7% at final follow-up.

**Conclusion:**

The ultra-short Proxima cementless stem showed a survival of 93.7% at a mean 12.1 years, proving the USS concept to be durable and effective. The USS is best suited for primary THA in Dorr A and B bones where diaphyseal bone preservation for future surgery is a priority.

**Level of Evidence: IV** Retrospective review consecutive case series.

## Introduction

Primary total hip arthroplasty (THA) provides salutary improvements in patient outcomes. Surgical technique continues to evolve with the procedure increasingly performed in younger, active patients [1, 2]. Cementless stem fixation is preferred in young patients, who have increased cyclic demands where cemented fixation is less optimal (3, 4). However, revision of cementless stemmed implants can be challenging due to bone density loss, wear debris reactions, stress shielding, fracture, and chronic infection. Shorter stems have been advocated to preserve bone and limit tissue damage during subsequent revision. Taken further, ultra-short stem (USS) designs are implants that do not engage the diaphyseal cortex and are the penultimate in short stem design. However, there is concern that these implants are at higher risk for mechanical loosening from excess bending and rotational forces, limiting osseointegration.

One such USS design is the Proxima^TM^ stem (DePuy International Ltd, Leeds, UK), that incorporates several design features enhancing initial stability to allow osseointegration. It maintains most of the femoral neck and is of anatomic design maximizing fit and fill. Moreover, the Proxima has a unique lateral metallic flare loading the lateral metaphyseal cortical column while resisting varus tilt forces [5]. We report our midterm results of a series of Proxima USS THAs in younger patients. We hypothesize, this USS will provide osseointegration to endure long-term, in young patients with greater hip demands.

## Methods

Between August 2005 and March 2009, a selected consecutive series of 35 primary THAs were performed using the Proxima femoral stem in patients < 70 years. Patients with developmental dysplasia or acquired hip deformity were excluded. Otherwise, the inclusion criteria were end-stage coxarthrosis in patients who failed non-operative treatment and returned for follow-up care. Subjects who died or were lost to follow-up before 10 years were excluded in the final follow-up analysis, which excluded two patients (3 THAs). One patient (2 THAs) died at 47 months, and a second patient was lost to follow-up at 74 months. Twenty-six patients (32 THAs) met the criteria for the study. This study received IRB approval prior to its initiation, and consent was obtained from all patients for participation in the study.

**Fig. 1:**
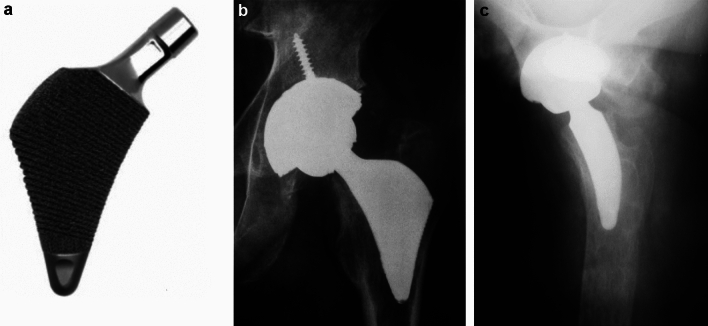
**a**–**c** Images of Proxima ultra-short stem. **a** Photograph of Proxima stem. Note the lateral coronal flare, which creates a symmetrical wedge design in the AP plane. The majority of the stem is coated with a porous coating and covered with hydroxyapatite. **b**–**c** Radiographs of the Proxima stem at 11.5 years. Note on AP view **b** how the lateral flare engages the lateral metaphyseal cortex, and the stem tip does not engage into the femoral diaphysis. Lateral view **c** again shows no diaphyseal engagement.

The Proxima stem (Fig. 1a, b) was made of forged titanium alloy, with an extensive circumferential porous coating layered on top with hydroxyapatite (HA) that was placed upon an underlying radial stepped macrotexture surface. The HA thickness was 30 µm. The stem was 64 mm long (larger sizes growing proportionally) and designed with an anatomic biplanar wedge. The distal taper tip did not engage the femoral diaphysis. The non-modular neck was a type 1 taper with a built-in 5-degree anteversion with standard and high-offset options. The Proxima design had a flare engaging the lateral metaphyseal cortex. This required aggressive lateral bone preparation with broaches.

Three cementless cup designs were used. These included the Duraloc™ (DePuy, Leeds, UK), the Pinnacle™ (DePuy International Ltd), and the ASR™ (DePuy, Warsaw, USA), a monobloc cobalt-chrome (CoCr) cup for large-diameter metal-to-metal bearing articulations. Ceramic, metal and polyethylene inlay inserts were used for the Pinnacle cup. Polyethylene inserts were used for the Duraloc. All heads (except for the ASR) were 28 mm in diameter.

Enrolled subjects returned for clinical evaluation postoperatively at 4 weeks, 3 months, 6 months, 1 year, and bi-annually thereafter. Any complications in treatment or return to the operating room were recorded. AP and lateral radiographs were taken postoperatively and at scheduled follow-ups. Patient functional outcomes were assessed before surgery, postoperatively at 3 months, 6 months, and bi-annually thereafter. Outcomes were assessed using the Harris Hip Score (HHS) [6].

### Surgical Technique

Anesthesia was standardized, using a neuraxial block (epidural or spinal). A 2nd generation cephalosporin (Cefuroxime 1.5gm) was administered perioperatively for 24 h. The patient was positioned in the lateral decubitus position and secured using the Peg Board positioner (Innovative Medical Products, Plainville, CT, USA). All surgeries were performed by the first author (SRT) utilizing a postero-lateral approach without fluoroscopy or computerized assistance [7, 8].

**Fig. 2:**
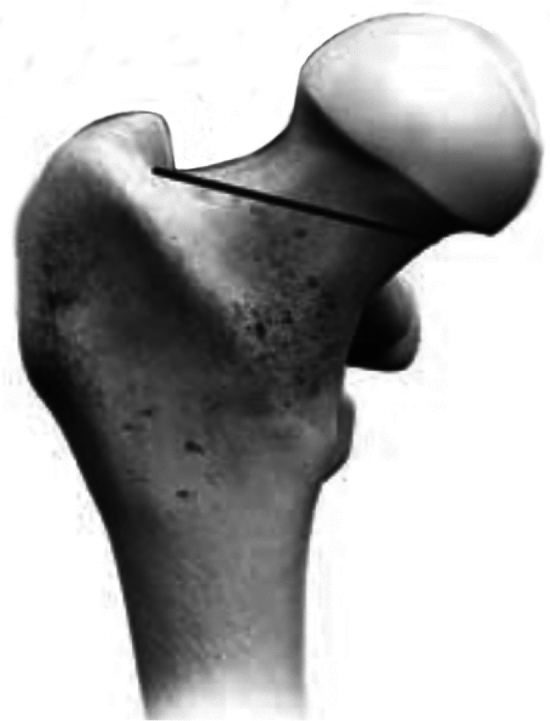
Diagram showing the position of the proximal femoral neck cut. The neck cut, in concept, was made (as close as possible) perpendicular to the femoral medullary axis. The cut starts medially at the subcapital junction, ending at the lateral neck as it blends into the greater trochanter.

**Fig. 3:**
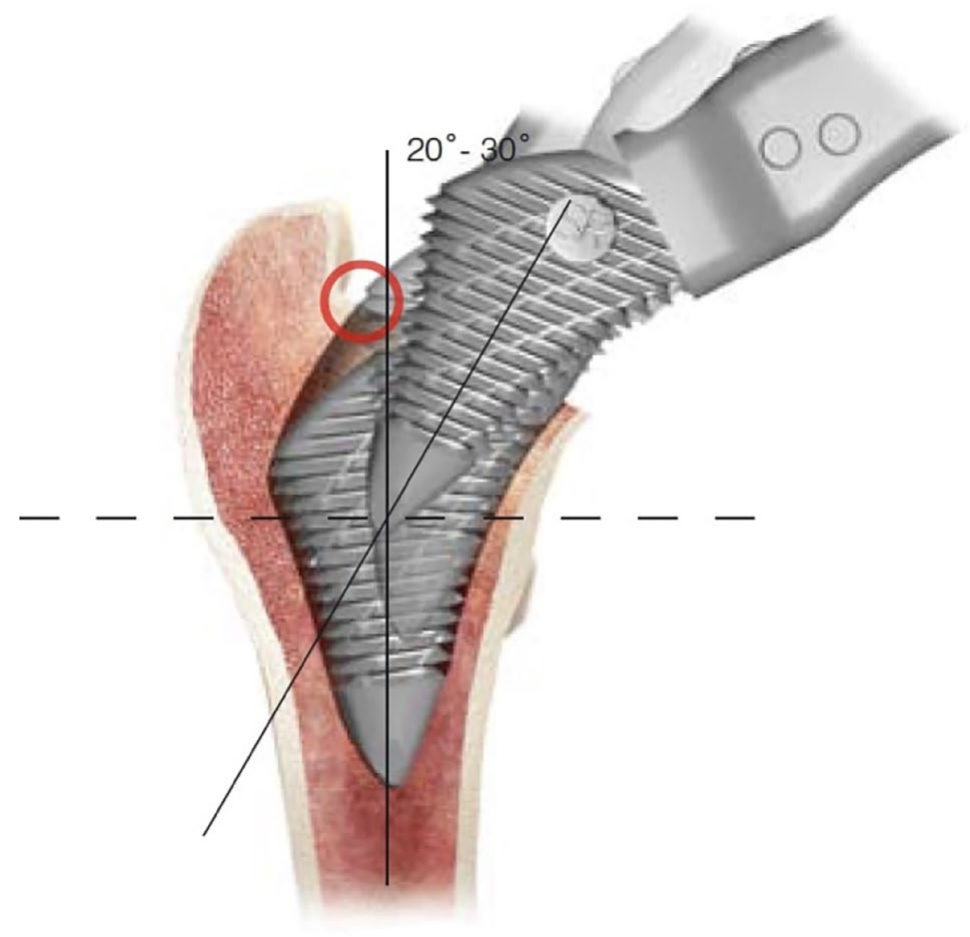
Broaching technique for the Proxima stem. The greater trochanter overhangs the femoral medullary axis proximally. In order for the lateral metaphyseal flare of the Proxima to reach under the greater trochanter, broaching and insertion must start at an angle reaching a position parallel to the femoral medullary axis. The red circle shows where the lateral femoral cortical neck must be removed to insert the stem into neutral axial alignment.

The femur was prepared first, and the final trial broach was left in place to protect the femoral neck during acetabular preparation. The femoral neck cut was unique, being nearly perpendicular to the femoral diaphysis (Fig. 2). Broaching was focused on preparing the lateral metaphysis to incorporate the lateral implant flare, demonstrated in Fig. 3. Initial broach entry followed the curve of the medial neck with emphasis on all impaction force directed laterally and distally. The implant position was confirmed with an attached axial femur guide on the broach. This was to ensure that the long axis of the femoral implant was parallel to the femoral diaphyseal axis, limiting varus tilt. The correct implant size required medial neck and lateral cortical column contact.

Acetabular preparation was performed with hemisphere reamers followed by shell trialing. Hip trialing was performed using modular neck and heads assessing hip stability and leg lengths, making adjustments as needed. Hips were closed in a layered fashion with absorbable sutures without a drain. Physiotherapy was started shortly after surgery with patients mobilized weight bearing as tolerated with an assist device. All were advised to avoid cross-legged sitting and ground-level activities for 6 weeks.

### Radiographic Analysis

Radiographs were independently analyzed by 2 independent physicians: an orthopedic arthroplasty fellow not associated with this series, and a musculoskeletal radiologist. Preoperative x-rays were graded for femoral bone type using the Dorr classification [9]. Radiographic stem positioning and implant fit were assessed at 3 months. The AP hip radiograph was used to measure coronal plane alignment (neutral, varus, and valgus), determined as the angle between the femoral anatomic axis and a line passing through the center of the implant. Neutral implant tilt was recorded as zero, varus tilt as minus, and valgus tilt as positive. The implant was considered misaligned if the tilt deviated greater than 3 degrees [10]. Metaphyseal fill was calculated as the ratio of implant width to canal width measured at the proximal border of the lesser trochanter in anteroposterior and lateral radiographs. Intramedullary fill was assessed by the implant/femoral canal ratio, which should be > 80% in the coronal plane and > 70% in the sagittal plane. A fill below these values was considered undersized [11, 12]. Radiographs at the latest follow-up were evaluated for implant stability and osseointegration. The modified Gruen method graded radiolucent lines around the implant on AP and lateral radiographs [13, 14]. Implant stability and fixation were assessed using the Engh grading [15, 16]. These criteria classified implant fixation as grade A-stable bone ingrowth, grade B-stable fibrous ingrowth and grade C-unstable.

### Statistics

Statistical analysis of recorded data was performed using SPSS® for Windows (Version 21.0, SPSS Inc., Chicago, USA). Stem survival was analyzed using Kaplan–Meier analysis, with implant revision as the endpoint. Preoperative and postoperative HHS were compared using a two-tailed t-test, with a p-value of < 0.05 considered significant.

## Results

The study comprised 32 THAs in 26 patients. There were 18 males and 8 females. The mean age was 47.9 years (range 28–67 years). The mean duration of follow-up was 12.1 years (range 10.8–13.8 years). The indications for surgery are listed in Table 1. Seven (21.8%) patients had Dorr type A bone, 18 (56.4%) had Dorr type B and 7 (21.8%) had Dorr type C femoral bone. The mean preoperative HHS was 48.5 (standard deviation (sd) = 10.2). The mean postoperative HHS at the 10-year evaluation was 87.8 (sd = 11.3). The mean improvement in HHS was 39.3 points (*p* = 0.0001). Table 2 shows the radiographic implant alignment measured at 3 months postoperatively. Twenty-two stems had a neutral alignment, 8 had a varus alignment, and 2 had a valgus alignment. No stems were misaligned. In modified Gruen scoring, 29 of the 30 stems reaching final follow-up had no observed radiolucent lines (RLLs). One patient had RLLs in zones 1 and 2. Coronal stem fill averaged 94% (range 87–98%), while sagittal stem fill averaged 87% (range 80–92%). No stem was underfilled. Table 3 reports the Engh grading for stem fixation for the 30 stems reaching final follow-up; 29 had Grade A fixation, and 1 had Grade B fixation.

**Table 1:** Hip condition requiring THA

Etiology	Frequency (%)
Avascular necrosis	20 (62.5%)
Inflammatory arthritis	5 (15.6%)
Osteoarthritis	4 (12.5%)
Post Traumatic arthritis	3 (9.3%)
Total	**32 (100%)**

**Table 2:** Post-operative stem alignment at 3 months

Stem alignment	Frequency (%)
0	22 (68.8)
–1	3 (9.3)
+ 1	2 (6.3)
2	3 (9.3)
–3	2 (6.3)
Total	** 32 (100)**

**Table 3:** Engh stem fixation grades at 10-year follow-up

Engh Grade	Frequency (%)
A (stable bone growth)	29 (96.7%)
B (stable fibrous growth)	1 (3.3%)
C (unstable)	0 (0%)
Total	**30 (100%)**

**Fig. 4:**
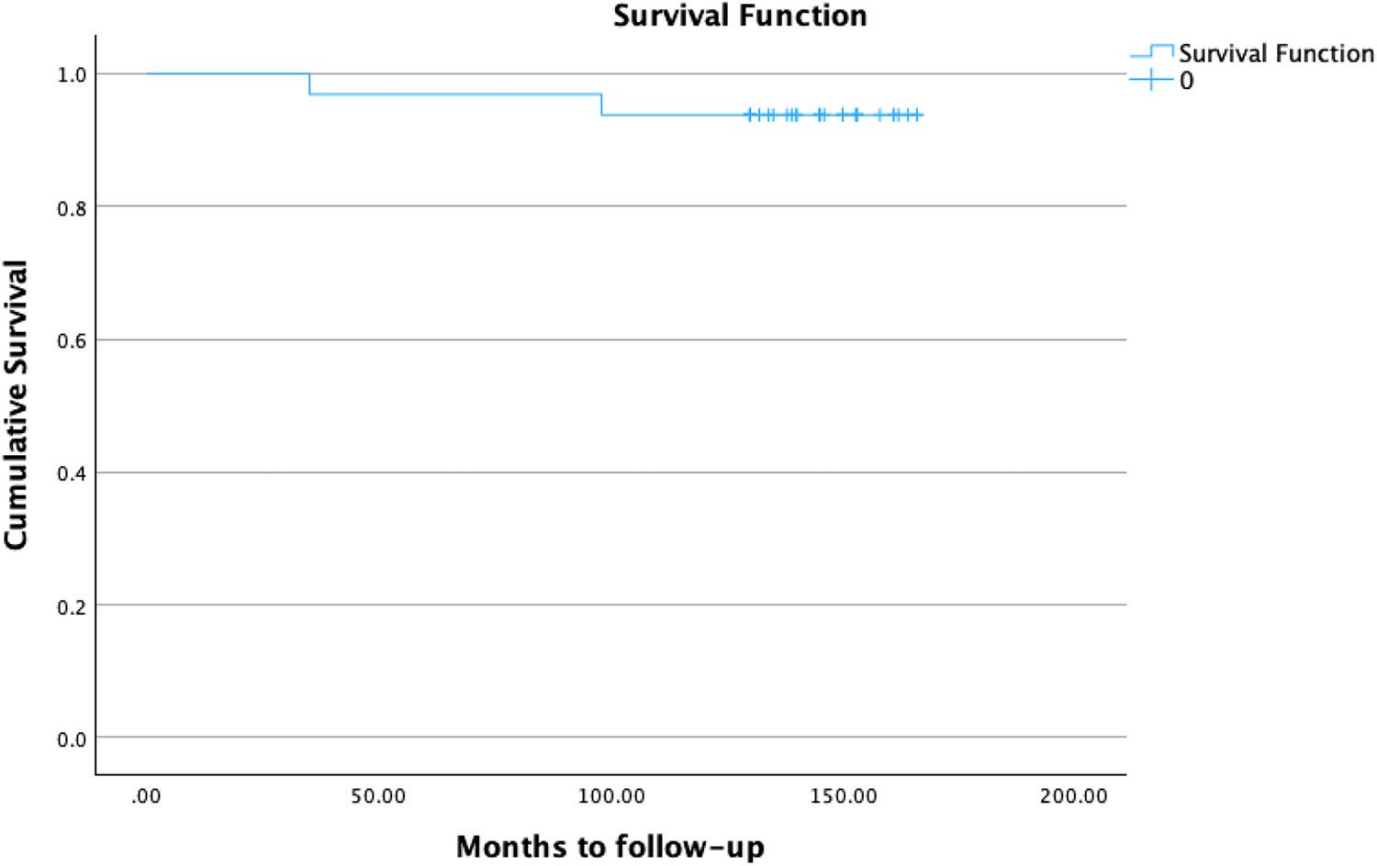
Kaplan–Meier survival curve of the Proxima stem shows a cumulative survivorship of 93.7% at a mean follow-up of 12.1 years. Stem revision was defined as the endpoint.

**Fig. 5:**
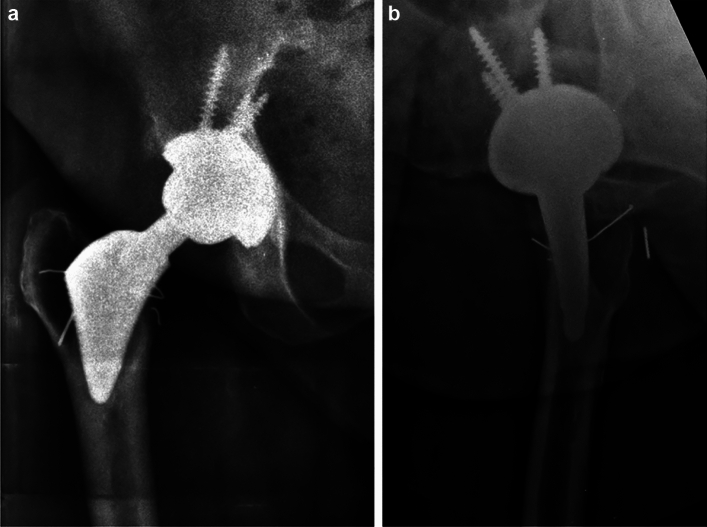
**a**,** b** Radiographs of Proxima at 13.5 years, performed for post-traumatic arthritis. **a** AP view demonstrating circumferential stem osseointegration. Note the preservation of proximal metaphyseal bone density. **b** Lateral radiograph demonstrating circumferential osseointegration and positive metaphyseal bone remodeling.

There were 2 cases of stem failure. The two loose stems had RLLs in all 14 zones, and both stems showed subsidence (3mm and 10mm). The first failure was associated with a periprosthetic joint infection (PJI) that occurred 3 years after surgery. The patient was treated with a successful 2-stage exchange. This patient had a diagnosis of inflammatory arthritis and a Dorr C femoral bone type. This patient showed early radiographic stem loosening before the onset of PJI, which occurred from a late hematogenous infection from urosepsis**.** The second failure was aseptic and revised at 8 years. Operative findings showed significant metallosis, lysis and loosening of the acetabular and femoral components. The patient had a diagnosis of inflammatory arthritis, Dorr C femoral bone type and a metal-on-metal ASR hip system. Figure 4 displays the Kaplan Meir survival curve, showing an overall stem survivorship of 93.7% (30/32 stems) at a mean 12.1-year follow-up. Figure 5 shows a case with a 13.5-year follow-up.

Four additional isolated acetabular revisions were performed in this series. All revisions were performed on the large-diameter metal–metal-bearing ASR cup. The revisions were performed for adverse metal debris reactions, with all patients experiencing pain and localized hip swelling. In these cases, the femoral stem was confirmed to be well fixed at the time of surgery and retained.

## Discussion

Cementless short femoral stems with limited diaphyseal extension have been utilized since the late twentieth century [17]. Jasty et al. showed that the function of the diaphyseal stem was superfluous after achieving proximal osseointegration [18]. Because of this observation, femoral stem design has evolved to lessen diaphyseal stem length by providing rotational and axial stability with proximal wedge designs. An ultra-short stem (USS) is defined as an implant that does not engage the diaphyseal cortex and is best described as a “stemless” implant. It is the penultimate design of short stem technology. A USS design brings the wedge loading of the stem above the level of the lesser trochanter. This provides advantages: loading is metaphyseal preserving proximal bone density, and second, by leaving the diaphysis undisturbed, subsequent revisions may be less intrusive and damaging.

The Proxima stem is a modified ultra-short, neck-sparing implant. In the classification of short femoral stem designs, the Proxima is a metaphyseal engaging neck-sparing stem with a lateral metaphyseal flare (JISRF–Joint Implant Surgery Research Foundation class 2-B) [19]. The lateral metaphyseal flare is unique, developed by F.S. Santori in Italy, starting with a series of custom design stems beginning in 1995, culminating in the eventual design of the Proxima stem for commercial release in 2005 [20]. The lateral flare resists varus stem tilt benefiting osseointegration. Finite elemental studies and clinical DEXA (Dual Energy X-ray Absorptiometry) analysis affirm this design concept [21]. Stem manufacturing was discontinued in 2013, with the remaining stems sold until depletion. The reason for discontinuation was a business “rationalization” to streamline manufacturing to a core femoral implant family after DePuy was acquired by Johnson & Johnson. Approximately 10,000 Proxima stems were inserted globally (personal communication EJM with DePuy International) [22].

Table 4 reviews all Proxima USS hip studies in the literature showing stem survivorship, all above 94%. This study is unique, being the longest mean follow-up to date performed by a surgeon outside of the Proxima design team and evaluators. Our stem survivorship was 93.7% at a mean follow-up of 12.1 years, similar to other Proxima series. Even though this stem has been discontinued, this study does provide insight into USS technology. First, our study targeted younger patients regardless of Dorr bone class. The distribution of A, B, and C bone types followed a bell curve. Our strategy was to use the USS in all bone types to avoid diaphyseal bone engagement. We believed this would mitigate metaphyseal stress shielding, and leave the diaphysis undisturbed, allowing a more simplified stem revision if needed [23].

**Table 4:** Summary of all published Proxima stem studies*

Author	No. of patients, No. of THAs	Mean age at surgery	Mean follow-up	Radiographic loosening	All cause stem survivorship
Santori FS et al*.* 2006 [26]	111 patients, 131 THAs	51 years (range 21–71)	5.3 years (range 3–11)	None	100%
Santori N et al*.* 2006 [5]	10 patients, 10 Proxima vs 10 short stems	58.5 years (range 37–64)	2 years (no range)	None	100%
Santori FS et al*.* 2010 [27]	109 patients, 129 THAs	51 years (range 21–71)	8 years (range 4.9–14.1)	None	100%
Ghera S et al*.* 2009 [28]	65 patients, 65 THAs	70.1 (range 46–87)	1.7 years (range 1–3.5)	None	100%
Tóth K et al*.* 2010 [29]	35 patients, 41 THAs	49 years (range 35–60)	2.2 years (range 1.1–3.6)	None	100%
Logroscino G et al***.*** 2011 [30]	19 patients, 19 Proxima vs 12 Nanos	50.2 years (range 37–64)	1 year (no range)	None	100%
Necas L et al*.* 2023 [31]	73 patients, 97 THAs	47.4 years (range 23–63)	8.8 years (range 5.2–14)	2%—no revisions	98.9%
Hrubina M et al*.* 2024 [32]	68 patients, 75 THAs	48.4 years (range 18–66)	9.5 years (range 8.2–15.7)	None	100%
Melíšik M et al*.* 2021 [33]	17 patients, 17 THAs	45.0 years (range 25–60)	9.4 years (range 5.3–12.3)	11.8%—no revisions	94.1%
Kim et al. 2011 [34]	84 patients, 84 THAs	78.9 years (range 70–88)	4.6 years (range 4–5)	None	100%
Kim et al. 2011 [35]	50 patients, 60 Proxima vs 60 Standard	54.3 years (range 26–77)	3.3 years (range 3–4)	None	100%
Kim et al., 2012 [36]	70 patients, 70 Proxima vs 70 conventional	74.9 years (range 50–94)	4.1 years (range 2–5)	None	100%
Kim et al., 2012 [37]	126 patients, 144 THAs	53.9 years (range 26–65)	4.5 years (range 4–5)	None	100%
Kim et al., 2016 [38]	201 patients, 221 Proxima vs 530 Short THAs	53 years (range 21–63.9)	12.3 years (range 10–13)	None	99.5%
Kim et al. 2016 [39]	200 patients, 200 Proxima vs 200 conventional–Bilateral THAs	53 years (range 26–54)	11.8 years (range 10–13)	1%—1 stem revised	99.5%
Mahmoud et al., 2017 [40]	25 patients, 28 THAs	51.4 years (range 16.7–68.2)	6 years (range 5.4–6.6)	None	100%
Melíšik et al., 2021 [41]	121 patients, 130 THAs	45.5 years (No range)	9.8 years (range 8–13)	1.5%—no revisions	100%
Choi et al., 2016 [42]	47 patients, 56 THAs	54 years (range 26–77)	4.6 years (range 4–5)	None	100%
Rastogi et al. 2016 [20]	41 patients, 50 THAs	45 years (range 35–55)	4.1 years (range 3–6)	2.4%—stem revised	97.6%
Malhotra et al., 2016 [43]	30 patients, 33 THAs	30 years (range 25–40)	6.5 years (range 5–8)	None	100%
Gombár et al., 2019 [44]	81 patients, 86 THAs	50 years (range 32–65)	9.3 years (range 7–11.6)	1.2%—revised	98.7%

^*^Other reasons for revision were excluded. Only cases where the stem was revised were included above

Second, we believed younger patients would have successful fixation regardless of Dorr bone type if meticulous stem preparation was performed. For the most part, our results did support this idea. Not unexpectedly, our two stem failures did occur in Dorr C bone (28.5% C bone failure rate), a bone condition that less favorably supports robust osseointegration. This is in contrast to Kim et al. who believed that the Proxima stem is acceptable in Dorr C bone type [24]. Based on our results, we feel the Proxima should not be used in Dorr C bone. One of the two failed stems also had a late PJI, but the stem subsidence occurred in the radiographic evaluation before the hip became infected. We advocate USS use in Dorr A and Dorr B bone if technical preparation provides optimal alignment, fit, and implant stability. To this point, our radiographic review showed all stems had acceptable alignment and fill, with 94% aligned within two degrees of the femoral medullary axis and 100% of stems meeting the criteria for optimal fill in coronal and sagittal planes. USS preparation requires meticulous surgical technique, avoiding stem mispositioning which is essential for durable osseointegration.

This report has several strengths. First, this series was conducted by a single surgeon experienced in hip arthroplasty and not affiliated with the implant design or initial rollout trials. This selects out designer bias. It also shows that USS technology can be utilized successfully by the general orthopedic community. What we consider a study strength is the selection of various acetabular cups and bearings. Those cases using large head metal–metal bearings showed decreased survival, requiring 5 cup revisions. However, the fixation of the USS was still robust and stem revision was not required. This was a unique observation of this study. Finally, this study has midterm follow-up, proving that osseointegration of the USS Proxima is durable.

There are several study weaknesses. First, this study was selective, choosing relatively young patients who can comply with post-operative restrictions, giving advantage to USS’s success. The effectiveness of USS technology would be better assessed by enrolling all patients intended for primary THA. Second, the study cohort was small. A larger powered cohort would be required to validate stem survival. Thirdly, this study was a single surgeon experience. A realistic performance curve would be best shown with a large series conducted by multiple hip arthroplasty surgeons of varying experience. Finally, the study would have more impact evaluating the USS concept in a randomized multicenter trial against a diaphyseal engaging stem with a similar proximal metaphyseal design employing a single acetabular cup and bearing. Although small in sample size, our findings provide valuable learning points in USS concepts that can be applied to future implant design.

Finally, we point out that the USS Proxima design does have technical shortcomings. First, because the stem requires a robust fit and fill, the surgeon cannot accommodate anatomic variances of the proximal femur and thus, stem version cannot be adjusted. Secondly, it is not possible to insert the Proxima stem into femurs with long femoral necks with a high valgus neck–shaft angle. In this anatomic variant, the femoral head center is superior to the greater trochanteric tip. To fit the stem into the lateral metaphysis, a very low neck cut is required, which significantly shortens hip length. An extra-long femoral head length may not make up for the shortening. Furthermore, compensating with an extra-long femoral head can excessively increase hip offset, causing lateral trochanteric pain and increasing the risk for a taper corrosion reaction [25]. These issues should be considered when selecting a USS for THA.

## Conclusion

The Proxima USS cementless stem achieved a 93.7% survivorship at a mean 12.1-year follow-up in patients with coxarthrosis with a mean age of 47.9 years. For successful osseointegration, the surgeon must ensure complete metaphyseal implant fill and optimal axial alignment. We recommend avoiding the use of USS designs in Dorr C bone.

## Data Availability

All data pertinent to current cases has been presented as a part of this manuscript.
